# Designing Effective Warnings about Addiction on the Patient Information Leaflet of Over-the-Counter Codeine Sold in England to University Students

**DOI:** 10.3390/ijerph17155490

**Published:** 2020-07-29

**Authors:** Jianan Zhao, Yun Chen, Ting Han, Stephen Westland

**Affiliations:** 1 School of Design, Shanghai Jiao Tong University, Shanghai 200240, China; jzhao2@cca.edu; 2 School of Design, University of Leeds, Leeds LS2 9JT, UK; sdyc@leeds.ac.uk (Y.C.); S.Westland@leeds.ac.uk (S.W.)

**Keywords:** patient education, medication management and safety, university students, codeine, patient information leaflet, over the counter

## Abstract

(1) Background: The harm of misusing over-the-counter (OTC) codeine-containing medicines among university students in England is being increasingly recognized. Based on English university students, this paper aims to study the importance of information design on information communication, explore methods for effective warning design, and investigate university students’ perception of OTC codeine. (2) Methods: The effective warning design is addressed through case studies, answering correctness by the heat map generated from the eye-tracking experiment (ETE), and the total time spent on the tasks. User perceptions are made though online surveys. (3) Results: Information design significantly affects the way user processes information. Therefore, two emphasized warnings displayed in the headline, and the “possible side effect (PSE)” sections and warning signs of addiction presented under the PSE are suggested as effective ways to display warnings. For students’ perception of OTC codeine, 80% of university students are unfamiliar with the substance. After reading the patient information leaflets (PILs), 47% recommended tight regulation on codeine. (4) Conclusions: The misuse of OTC codeine could be a potential problem among English university students. The design of the PIL significantly influences the chance of unintentional medicine misuse. The display of warnings on the PILs of OTC codeine should be redesigned for better understanding.

## 1. Introduction

With the potential harm of over-the-counter (OTC) codeine being increasingly acknowledged, a regulation on OTC codeine-containing analgesics was implemented in 2014. A warning term “Can cause addiction. For three days use only” and the warning signs of addiction were mandatory for every patient information leaflet (PIL) of OTC codeine-containing analgesics. However, without a further indication of where and how to display warnings about addiction, the effects of this regulation remain uncertain and limited [1,2].

OTC medicine is usually considered safe to use and available for self-medication [1,3]. Even though OTC medicines are comparably safe and mild in effects, the misuse of medicine, which was proven not rare in the UK, can still lead to severe consequences such as addiction and fatality [4,5,6]. This situation has raised concern since medicines such as OTC codeine, the most widely used opioid medicine, is very likely to cause harm [7,8,9]. Codeine, an opioid pain reliever, is available on prescription, or in lower-strength OTC medicines as a companion to other ingredients such as ibuprofen, caffeine, and paracetamol since it is useful in extending the duration of effects [10,11,12,13,14]. However, the misuse and abuse of OTC codeine-containing medicines can lead to not only addiction caused by codeine but can also lead to renal and liver damage caused by ibuprofen and paracetamol [11,15,16,17,18]. Besides, the consumption of codeine could lead to long-term use and dependence on opioids or other addictive substances such as heroin [5,8,19,20,21,22].

Codeine misuse and abuse are potentially harmful to individuals, societies, and the economy. Codeine misuse can be especially problematic for the United Kingdom since 44.2 tons of codeine was consumed in the UK in 2016, which was the second-biggest consumer of codeine in the world [21,23]. Among all English nationals, university students are at high risk of misusing OTC codeine, since the university students were proven more likely to misuse and abuse medicines compared to other adults [24,25]. However, insufficient attention was paid to drug intervention of English university students [26].

With increasing awareness of the severe outcomes of codeine misuse, interventions on the production and sale of codeine-containing medicines, including direct pharmacy intervention and online monitoring have been proposed and adopted in England [14]. However, it has been shown that a majority of people in England were not fully aware of the potential risks when they started using codeine-containing medicines [14,16,26]. Therefore, more efforts on patient education is needed, since patient education is effective in preventing medicine misuse [26,27,28,29,30,31,32].

For patient education, patient information leaflets (PILs) (a package insert that is mandatory for all medications sold in England) was rated the most reliable and essential source of side effects of medication [33,34]. Accordingly, a tightened regulation on the PIL of OTC codeine was published by the UK government in 2014 [35,36]. The term “Can cause addiction. For three days use only” and an indication on the signs of addiction is demanded for every PIL [2]. However, without further indications of where and how to display this information, the effects of this regulation remain unknown and are potentially limited. Under the circumstances where information design is found influential to the accuracy and amount of information acquired by readers, the investigation on the displacement of warnings about addiction is crucial for increasing public awareness and decreasing codeine misuse [37,38,39].

Therefore, utilizing a sample from English university students, the aims of the research were: (1) to investigate how essential the information design is for effective communication, (2) to identify an effective way to display warnings about addiction on the PILs of OTC codeine, and (3) to study English students’ attitudes and perceptions of OTC codeine-containing medicines.

To achieve these research aims, the research started with two case studies, one offline and one online, to assess the availability of OTC codeine among English university students and collect samples for the following experiment. Then an eye-tracking experiment along with a pre-experimental online survey and a post-experimental online survey was used to identify students’ perception of OTC codeine, the impact of information design on eye-movement, and effective ways of displaying warning terms. Two samples with similar word count, template, and efficacy but with different ways of displaying warnings were chosen for the eye-tracking experiment. Thus, the independent variables are the displayed location of warning information and appeared frequency of occurrence, and the dependent variables are participants’ reaction time and accuracy of finding several specific warning terms and attention locations. One sample was expected to establish a feasible standard for warning design, based on the understanding of information design principles [40,41,42,43]. We hypothesized that frequently appeared and highlighted information is more accessible for readers to recognize. With a systematic analysis of the research data and findings, this paper will conclude with answers to three research aims and the hypothesis proposed in the sample selection.

## 2. Materials and Methods

Three experimental methods were adopted. Two rounds of case studies on OTC codeine-containing medicines that are on sale were conducted to develop a better understanding of existing warning designs of addiction on the PILs. An eye-tracking experiment, which measures readers’ horizontal gaze nystagmus, was conducted to study how the information affects mental functioning. Finally, surveys about personal information and the perception of OTC codeine was conducted before and after the eye-tracking experiment.

### 2.1. Case Study

In the first round of case studies, PILs of 46 OTC medicines available in England were collected in hard copies from 16 English volunteer students at the University of Leeds. Among the PILS collected, 16 were PILs of OTC analgesics. Five of OTC analgesics contained codeine as one of the active ingredients, namely, Syndol Tables from SANOFI, Solpadeine Plus from Omega Pharma Ltd., Paramol Tablets from Reckitt Benckiser Healthcare Ltd., Co-damol Tablets from Accord, and Co-damol Effervescent Tablets from Zentiva.

Then, in the second round of case studies, more PILs of OTC codeine-containing medicines were collected online through the electronic medicines compendium (EMC), which is a website that offers more than 14,000 documents of medicines licensed for use in England [44]. The other 14 active PILs of codeine-containing medicines were found. The PILs would be investigated in terms of target symptoms, word count, structural layout, and placement of warning signs of addiction.

### 2.2. Eye-Tracking Experiment and Survey

#### 2.2.1. Sample Selection

For the eye-tracking experiment, two samples with different places and ways to display warning signs were selected from PILs of OTC codeine collected in the case studies. Based on our understanding of information design principles, one was expected to establish a feasible standard of information placement in comparison to another sample. In order to minimize the variance from wording and template, the selected samples were similar in word count, text column number, and efficacy. In accordance with these criteria, two PILs with a two-column layout, the PILs of Solpadeine Plus from Omega pharma Ltd. and Codeine Phosphate from Thronton & rose Ltd., were selected as Sample 1 and Sample 2, respectively. Based on information design principles [40,41,42,43], we hypothesized that the more frequently the information is mentioned, the more accessible the information is to find, and the terms highlighted in color or icons are more legible than using bolded words, followed by bolded sentences and then non-bolded sentences. Therefore, Sample 1 was assumed to establish a feasible standard for the way of displaying warning signs of addiction. Meanwhile, Solpadine Plus was also identified as the most common licensed OTC codeine in Europe [45].

#### 2.2.2. Questions in the Experiment

A pre-experimental survey was used to collect the user’s personal information and perception toward OTC codeine. Besides closed questions about age, gender, nationality, and the hometown of participants, two open-ended questions were designed to investigate participants’ perception of codeine-containing medicines.

Then, six tasks were taken by participants at the beginning of the eye-tracking experiment. Participants were asked to find task-related information on the leaflets. The first three questions required them to find information on target symptoms, dosage, and side-effects of the medicine, which were proven to be the most prominent information of a PIL [46]. The next three questions asked participants to identify why the medicine is only for a short-time use, the signs of addiction, and the frequency of mentioning the warning sign of addiction in the sample.

At last, a questionnaire for the follow-up survey was created to identify participants’ attitudes toward OTC codeine-containing medicines, and their perception of design. There were four open-ended questions and one question was asked twice, both at the beginning and the end of the experiment. In total, 16 questions were used throughout the experiment, which are summarized in Table 1.

#### 2.2.3. Participant Recruitment

In order to ensure similar cultural backgrounds and proficiency in English, we recruited 30 registered university students at the University of Leeds with English ethnicity and no specific restrictions on their major. The gender of the participants needed to be balanced since gender was relevant to substance abuse [47]. The participants were recruited online through an email written by the researcher and sent by the reception of the University of Leeds. This email contained the introduction of research background and objectives, the information of time and location, the target participants for the eye-tracking experiment and online surveys, and a link to a booking website (youcanbookme.com) which allowed the qualified participants to each select and book an experiment session online. In total, 30 qualified participants were recruited for the experiment. Participant recruitment and experiments took place from November to December 2019.

#### 2.2.4. Testing Procedure

The experiment was conducted using an eye-tracking device to record each participant’s eye movements for each sample. Researchers waited in the room ahead of the reserved session, with information sheets and consent forms prepared and printed out. As participants arrived, they were each introduced individually to the background, aim, and potential risks of the study through an information sheet and oral explanation by the researchers. The consent forms were then presented and signed before the commencing of the experiment.

For the implementation of the experiment, each participant was scheduled a maximum of one hour to finish a questionnaire, an eye-tracking experiment, and a follow-up survey. Once completing the pre-experimental survey, the eye-tracking experiment could be implemented. During the test, each participant was presented with the two samples in random order; that is, every 15 participants would be presented with samples in the same order. During the experiment, the participants were first given about 30 s to look at the sample before the tasks were introduced. Then the six different tasks of each sample were presented to the participants on the screen by order. They were asked to write down answers to each task on paper. However, participants were not allowed to go back to check answers and were instructed not to go back to check answers or write down answers based on their knowledge. This process was conducted again for the sample presented in the second order.

After completing the eye-tracking experiment, a follow-up survey was displayed online using Google Forms. Participants were asked to complete the questionnaire by themselves. Intervention and assistance from the researcher were only given when questions or concerns were bought up by the participants. Each participant was given five English pounds at the end of their session as thanks for their time and contribution.

#### 2.2.5. Data Collection and Analysis

The information collected from the case study was organized and analyzed by using SPSS (IBM, Armonk, NY, USA) statistics. As for the results of the eye-tracking experiment and online survey, all research data would be retained in the digital format. The results of the eye-tracking experiment were displayed in heat maps; the influence of information design on the effectiveness of the PIL would be measured based on the overlap of the correct answer and color-coded areas, the color-coded areas that did not contain the correct answer, and the sum of time to find information for all the tasks.

#### 2.2.6. Ethical Consideration

This research complied with the American Psychological Association Code of Ethics and was approved by the Institutional Review Board at the University of Leeds (LTDESN-116).

## 3. Results

### 3.1. Case Study

Five out of 46 PILs collected in the first round of case studies were codeine-containing medicines. They were offered by five different volunteers. Then, 14 leaflets of codeine-containing medicines (with 10 for pain relief, three for dry cough, and one for cold & flu) were found on the EMC by researching the keyword, “codeine,” within the category of OTC medicines. Therefore, 19 PILs of codeine-containing OTC medicines were found for further investigation. As shown in Table 2, the 19 PILs were grouped by efficacy and listed in alphabetic order. They were each analyzed in terms of text layout, word count, visual elements, and print size. As seen in Table 2, 78% of codeine-containing medicines were designed to treat pain. The content of codeine per pack varies with the name of medicines and pack size available on the market. As for the design of the PILs, 68% of medicines were designed with a two-column structure layout, and the warnings about addiction were not colored or did not accompany an icon in 63% of the PILs.

The ways to present a warning about addiction were investigated in detail. Four ways of displaying warnings about addiction were identified in four sections of the PILs. In Table 3, with the use of a heat map, four kinds of warning signs were presented in four different colors and were distributed in four rows, each row symbolizing a section of information on the PIL. As seen from Table 3, the warning terms and signs of addiction were presented in all codeine-containing analgesics, either highlighted or unhighlighted. However, for medicines targeting on cold and flu and dry cough, only one of them contained a warning about addiction.

**Figure 1:**
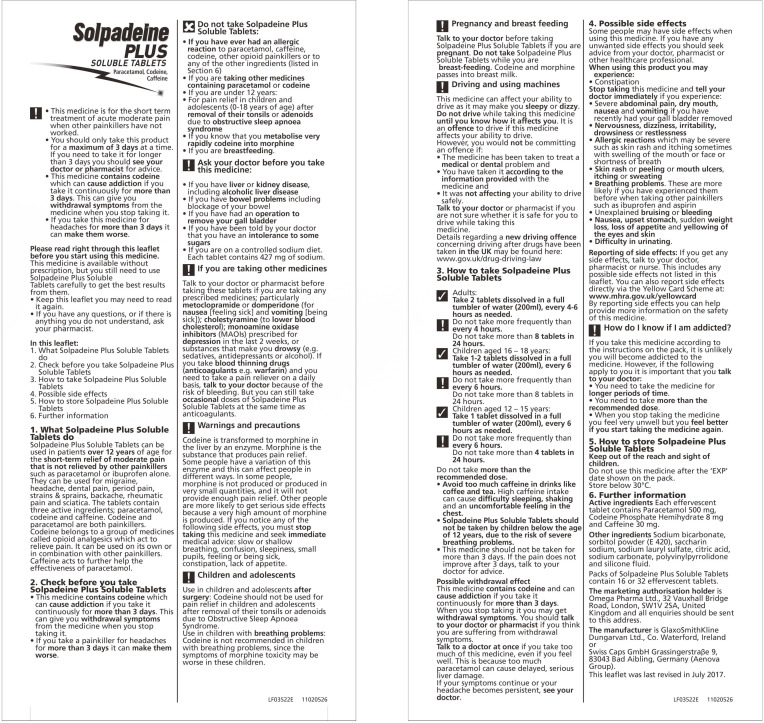
Sample 1 [48].

**Figure 2:**
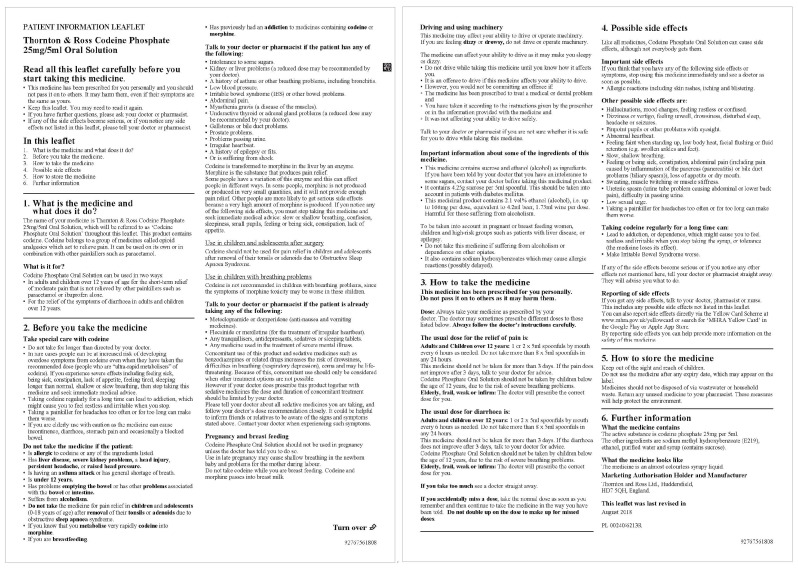
Sample 2 [49].

Based on the criteria for sample selection discussed earlier in the Section 2.2.1., two PILs with similar layout, word count, and efficacy but with a different way of displaying warnings about codeine were selected for further testing. As seen from Table 2 and Table 3, leaflet number 4 and 12 shared similar layout, word count, and efficacy, but most different way of displaying warnings of addiction. Therefore, the PILs of Solpadeine Plus from Omega pharma Ltd. and Codeine Phosphate from Thornton & rose Ltd. were selected as Sample 1 (Figure 1) and Sample 2 (Figure 2) [48,49], respectively.

### 3.2. Eye-Tracking Experiment

#### 3.2.1. Participants’ Information and Their Perception of OTC Codeine

**Figure 3:**
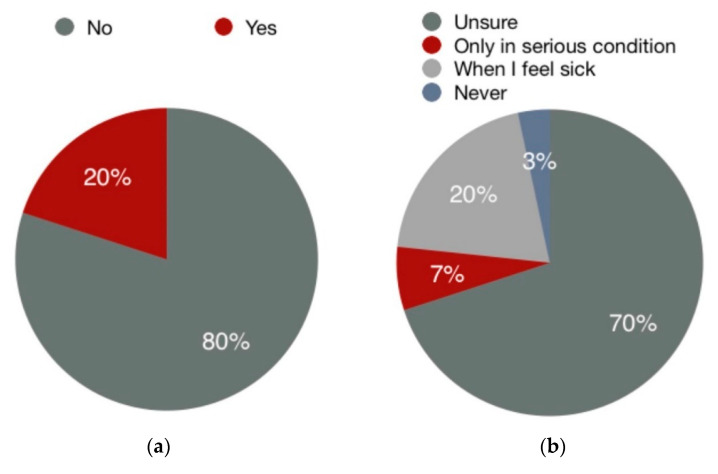
The results of two questions from the pre-experimental survey: (**a**) Are you familiar with opioid analgesic or codeine-containing medicines? (**b**) Please identify how many times the warning sign of addiction was mentioned in this leaflet.

Among the 30 participants of the eye-tracking experiment, 15 were female and 15 were male. Twenty-three participants were between the age of 20–30, the other seven were 30–40. All the recruited participants were English nationals, and five of them mentioned they were immigrants to England. English was the first language of 25 participants. The results for participants’ familiarity and attitudes toward OTC codeine are summarized in Figure 3. Twenty-seven percent of them tend to purchase codeine-containing medicines when feeling sick and merely 3% chose never to purchase it. Eighty percent of them were unfamiliar with codeine.

#### 3.2.2. Testing Results

**Figure 4:**
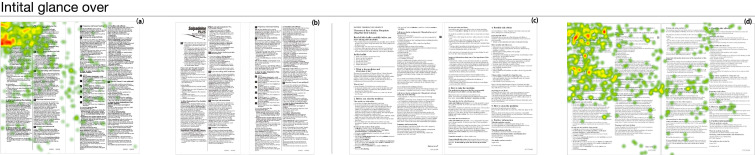
The result of the eye-tracking experiment for the first 30 s (shown by heat-map images); the warmer the color gets, the more the attention was gained in the section. (**a**) The field of view while looking at sample 1; (**b**) sample 1; (**c**) the fields of view while looking at sample 2; (**d**) sample 2.

**Figure 5:**
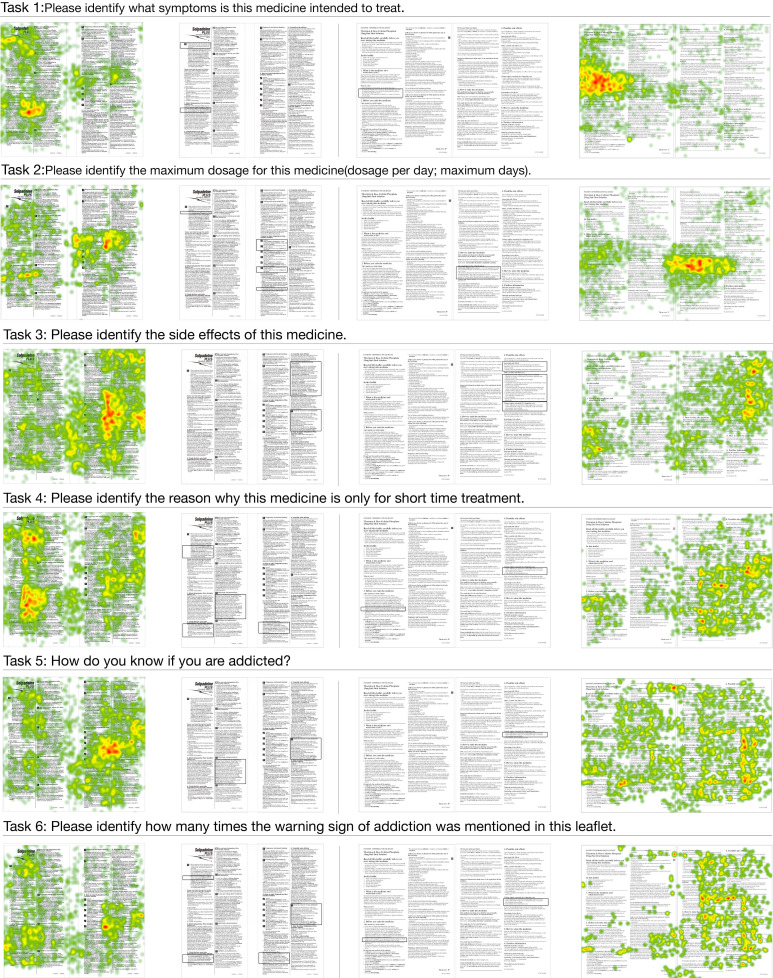
Field of view: the result of the eye-tracking experiment (shown by heat-map images); the left side is Sample 1 and the right side is Sample 2. The correct answers for each task are boxed in the figures.

**Figure 6:**
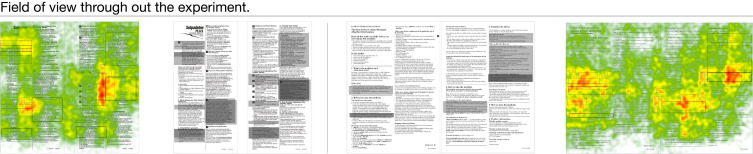
Field of view for all six tasks, the left side is sample 1 and the right side is sample 2. The correct answers for each task are boxed in the figures.

For the eye-tracking experiment of two previously selected samples, the six tasks of each sample were introduced to each participant during the experiment. The results of the eye-tracking experiment were summarized in Figure 4, Figure 5 and Figure 6 using heat-maps. Figure 4, Figure 5 and Figure 6 presented participants’ initial focus of the sample, the eye movement, and the coverage of eyesight throughout the whole experiment, respectively. The field of view was colored in the heat-map. The warmer the color gets, the better the attention was gained in the section. The correct answers for each task are boxed in the figures.

As shown in Figure 4, information placed on the upper left corner of a PIL was most likely to be observed in the first 30 s of glances.

Participants’ eye movements while doing six tasks are presented in Figure 5. For the first three tasks, participants tended to rely on the headline section, especially when there was a headline section that contained a summary of the information. Then for the fourth question on the reasons for short-time treatment, readers of Sample 1 were capable of finding the answers in two sections. The majority of them primarily found the information under the sub-heading, “warning and precaution” in bold font, while others found the information in the headline section. For Sample 2, even though the readers eventually found the information, the majority of them skimmed all over the places to seek information. In task 5, the signs of addiction were asked. This information was displayed twice in Sample 1 and once in Sample 2, respectively. Nearly all readers of Sample 1 identified the information in the sub-section called “How do I know if I am addicted?”, which was highlighted and labelled with an icon. For that of Sample 2, when the information was hidden in the “possible side effect” section, it seemed that the participants lost focus. For task 6 which asked about the frequency of displaying warnings about addiction in each sample, the result indicated that readers were capable of finding the warnings on multiple sections of Sample 1, while only one warning was found in Sample 2 by the majority of participants.

For the field of view presented in Figure 6, the intense color occupied 4.68% and 7.85% of the area in Sample 1 and Sample 2, respectively. Among the intensely colored area, 38.5% of the area in Sample 1 and 57.5% of the area on Sample 2 remained unboxed, which means the readers of Sample 2 spent more time on ineffective information than those of Sample 1.

**Figure 7:**
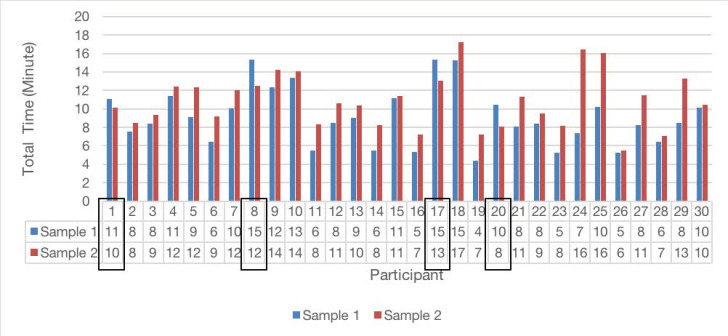
The time for each participant to find answers for all tasks by using different samples. Only four participants which are marked by a black box spent a longer time on sample 1 than sample 2.

Then, the total time used by each participant to find correct answers to the tasks was measured and summarized in Figure 7. The average time spent on Sample 1 was 9.1 min, and that on Sample 2 was 10.9 min. The *p*-value was calculated as 0.029. Therefore, information design could influence readers’ speed of finding information. In general, participants found information more quickly using Sample 1 than using Sample 2.

### 3.3. Interview

**Figure 8:**
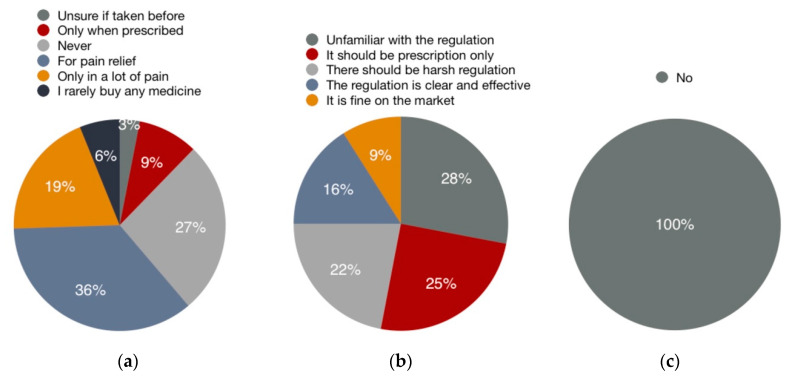
The results of two questions from a follow-up survey. From the left to the right: (**a**) In what situation do you tend to buy codeine-containing medicines? (**b**) How do you think of the availability and regulation of codeine? (**c**) Have you seen these two patient information leaflets before?

As shown in Figure 8, after being informed with the addictive risk of OTC codeine, 27% of participants said they would never buy codeine-containing medicines; 55% of participants were likely to purchase OTC codeine-containing medicines in the future, and six percent of them would only consume codeine-containing medicine if it was prescribed. For the availability and regulation of OTC codeine, 28% of the participants were unfamiliar with the regulation and danger; 47% participants argued that there should be tight restrictions on the regulation and availability since it had the potential for addiction, and 25% of them found that regulation and availability of OTC codeine was appropriate enough. None of the participants had seen Sample 1 and 2 before the eye-tracking experiment. Their preference for both samples was summarized in Table 4.

For participants’ preference of sample PILs summarized in Table 4, Sample 1 and 2 were both favored by 50% of readers. However, the feedback from the participants indicated that Sample 2 was mostly favored for its overall layout, while Sample 1 was preferred for its clear heading, summarization of information in the headline section, and clear and repeated warning on addiction.

## 4. Discussion

With a particular focus on English university students, this research aimed to explore the effect of information design on readers’ eye movement, ability to identify a legible warning design for the PIL, and to investigate university students’ perception of OTC codeine. Responding to the first research aim, the result proved that different ways and places of displaying a warning about addiction on the PIL significantly affected readers’ eye movement. Legible warning designs could speed up the time readers spent searching for information, thus promoting patient education and reducing unintentional medicine misuse [30,39,50,51]. For the hypothesis about warning design made prior to the experiment, the results showed that university students tend to read from the upper left and identify risk factors in the “possible side effect (PSE)” section, but minor differences were identified on how different formats of the highlight influenced their eye movement. Therefore, the proposed hypothesis was only partially correct. For the second research aim based on the research results, it is suggested that in order to achieve effective communication of warning, two distinctively highlighted warning terms should be displayed in both the headline section and the “possible side effect” section, and the warning signs of addiction should be placed in a separate sub-section under the “possible side effect” section. Then for the last research aim, which was about students’ perception of OTC codeine, a majority of English university students were unfamiliar with the risk of codeine. After acknowledging the risks of codeine through reading the PILs, 47% of them suggested tight regulations on OTC codeine, and 55% of them may purchase it in the future.

With the influence of information design to the effective communication of information being approved [38,46,52], findings of this research added credit to previous researches by using results of the eye-tracking experiment to present English university students’ ways of reading information on the PIL. Since information design was necessary for patient education [30], the effective presentations of warning about addiction were aimed to be identified in this research. For the navigation method, the result suggested that most readers tend to start reading the information from the upper-left corner. This finding proved the usefulness of the headline section; however, this result contradicted that of Dolk et al. [53]. When the information process model was identified by Lonsdale [54], the result suggested that people are more likely to scan (read the information thoroughly from up to down and left to right) the PILs that have a headline section which contained a summary of relevant information and skim (purposefully search for content) the PILs which were without such section. For the risk-related information, participants were more likely to search within the headline section that contained a summary of relevant information, and the “possible side effect” section. Therefore, the term, “Can cause addiction. For three days use only” displayed clearly both at the upper-left corner and in the “possible side effect” section of a PIL is the minimum requirement of the effective communication. For the signs of addiction, the result suggested that the best practice was to include the information in a highlighted sub-section under the “possible side effect” section. This finding is meaningful since the easiness to read is relevant to patient education and users’ willingness to read [55]. However, if this is used as the standard, then only 21% of collected PILs of OTC codeine met the criterion.

Apart from the results of the eye-tracking experiment, changes in students’ perception toward codeine also indicated the significance of a legible PIL. According to the findings, students became more conservative about codeine consumption because of education about codeine, as the rejection rate on codeine consumption raised from three percent to 27%. In total, 80% of participants were unfamiliar with codeine, demonstrating the importance of the warnings about addiction for patient education, especially for first-time users [13]. However, relevant warnings were not found in 15.7% of the recruited PILs of OTC codeine, especially for those not aimed at treating pain. This finding can be worrying since codeine was found in PILs of OTC analgesics offered by 31.3% of volunteers. Current regulations on warning signs of codeine-containing medicines are also actionable for OTC codeine-containing analgesics. Similar regulations may be applied to OTC codeine that treats other symptoms, such as dry cough, cold, and flu. Besides, another worrying finding was that half of the participants retained the possibility to consume OTC codeine in the future even though they were informed of the risk of codeine. This could be the reason for banning OTC opioids, which was suggested in previous studies and implemented in Australia in 2018 [56,57].

### Limitations

It should be acknowledged that this study was limited in several aspects. Firstly, the result could be potentially limited by the sampling strategy since the participants were only recruited within the University of Leeds. Secondly, the effect of the recommended ways of presenting the information could be influenced by multiple factors once it is applied in practice, such as literacy levels, co-morbidities, familiarity levels, and different design elements used in each PIL [58,59,60,61,62]. Thirdly, while this study only discussed the way of displaying warnings on the PIL with a two-column layout for health students, it might be essential to further prove the suggested way of displaying warning terms again by focusing on the PILs with a different design. Lastly, since better pain relief is one of the reasons for medication misuse [5], conducting the experiment again by testing patients who might benefit from OTC codeine could be another way to further verify the results.

## 5. Conclusions

In order to prevent OTC codeine misuse, this research proposed a more specified way to present warnings of addiction. It is suggested that there should be at least two highlighted terms, “Can cause addiction. For three days use only,” displaying separately in the headline section and “possible side effect” section of a PIL. The warning signs of addiction should be placed outstandingly as a sub-section within the section of “possible side effects.” Better patient education is meaningful for English university students since most of them are unfamiliar with codeine, which would help reduce the chances of unintentional codeine misuse.

## Figures and Tables

**Table 1:** Questions asked before, during, and after the eye-tracking experiment.

**Questions asked before the eye-tracking experiment:**	
Question 1:	How old are you?
Question 2:	Which gender do you identify yourself as?
Question 3:	What is your nationality?
Question 4:	Where is your home town?
Question 5:	Are you familiar with opioid analgesic or codeine-containing medicines?
Question 6:	In what situation do you tend to buy over-the-counter codeine containing medicines?
**Questions asked during the eye-tracking experiment:**	
Task 1:	Please identify what symptoms that this medicine is intended to treat.
Task 2:	Please identify the maximum dosage for this medicine (dosage per day; maximum days).
Task 3:	Please identify the side effects of this medicine.
Task 4:	Please identify the reason why this medicine is only for short-time treatment.
Task 5:	How do you know if you are addicted?
Task 6:	Please identify how many times the warning sign of addiction was mentioned in this leaflet.
**Questions asked after the eye-tracking experiment:**	
Question 1:	In what situation do you tend to buy over-the-counter codeine containing medicines?
Question 2:	How do you think of the availability and regulation of over-the-counter codeine?
Question 3:	Have you seen these two patient information leaflets before?
Question 4:	Which patient information leaflet is more helpful to you? Why?

**Table 2:** Analysis of the patient information leaflets (PILs) of over-the-counter (OTC) codeine containing medicines collected for case studies.

No	Medicine	Efficacy	Text Column	Word Count	Icons & Colour	Print Size/cm
1	Co-damol Tablets (Accord)	Pain	2	1659	None	21.0 × 29.6
2	Co-damol Tablets (M&A Pharmachem Ltd.)	Pain	2	2189	None	12.4 × 20.7
3	Co-damol Effervescent Tablets (Zentiva)	Pain	1	3020	None	20.9 × 50.0
4	Codeine Phosphate (Thornton & rose Ltd.)	Pain	2	2068	None	21.0 × 29.7
5	Codis 500 (Reckitt Benckiser Healthcare UK Ltd.)	Pain	2	1788	None	24.1 × 48.2
6	Migraleve Film-coated Tablets (McNeil Products)	Pain	3	2478	Both	25.0 × 14.5
7	Nurofen Plus (Reckitt Benckiser Healthcare Ltd.)	Pain	2	4914	None	19.2 × 29.6
8	Panadol Ultra Tablets (GlaxoSmithKline Healthcare)	Pain	2	2152	Icon	14.1 × 10.6
9	Paramol Tablets (Reckitt Benckiser Healthcare Ltd.)	Pain	2	1839	None	18.0 × 26.7
10	Paracetamol, Codeine & Caffeine (Fannin Limited)	Pain	2	1717	None	29.7 × 23.8
11	Paracodol Tablets (Delpharm Gaillard)	Pain	2	1468	Icon	15.0 × 25.0
12	Solpadeine Plus (Omega pharma Ltd.)	Pain	2	1936	Icon	15.0 × 30.5
13	Syndol Headache Relief Tablets (SANOFI)	Pain	2	1894	None	21.0 × 28.0
14	Syndol Film-Coated Tablets (SANOFI)	Pain	2	2897	None	19.0 × 29.7
15	Veganin (Omega Pharma Ltd.)	Pain	2	2482	Colour	14.0 × 22.9
16	Pulmo Bally (DDD Limited)	Cold & flu	3	1048	None	16.5 × 12.0
17	Codeine Linctus (Thronton & rose Ltd.)	Dry cough	3	1196	Icon	17.0 × 6.49
18	Codeine Linctus BP (Pinewood Laboratories Ltd.)	Dry cough	1	1320	None	21.0 × 29.7
19	Galcodine Linctus (Thronton & Ross Ltd.)	Dry cough	4	1300	Icon	21.0 × 29.7

**Table 3:** Investigating the presentation for warnings of addiction.

Presenting Warnings of Addiction	1	2	3	4	5	6	7	8	9	10	11	12	13	14	15	16	17	18	19
Headline section																			
“Before use” section																			
“How to take” section																			
“Side-effect” section: (“How do I know if I am addicted”)																			
	Labeled with color or icon:																		
	Labeled in bolded words:																		
	Labeled in bolded sentences:																		
	Labeled:																		
	Unlisted:																		

**Table 4:** Answers to the question “Which PIL is more helpful to you?”.

	Frequency	Feedbacks	Frequency
**Sample 1**	15	I felt that I could find things easier in sample 1	2
		Clear layout, clear heading, clear warning, summarized the important information at the top of the sheet.	4
		Easier to find the issue as they had a bold format. Address issues more.	2
		The structure of the pamphlet, notable the subheadings and bullet points.	1
		All the primary information was right at the start of the leaflet which you immediately go toward. The other one you have to hunt for the information.	1
		Clear to read, and the font was larger. It is easy to find what you needed with subheading in comparison to the second.	2
		It was clearer in pointing out the addictive property of codeine, which is very important and the information was grouped together well within the subtitles.	1
		Sample 1 had more information about addiction, and it was clearly laid out.	2
**Sample 2**	15	The categories are divided better into sections.	1
		The layout and typeface are more legible than Sample 1.	8
		It was easier to read. Sample 1 had too much bold lettering.	1
		This one is easier to read. The information on Sample 1 is all over the place.	1
		The headings are more descriptive of each section.	2
		It has more subheadings than Sample 1.	1
		Sample 1 had too much information; the font size is too small to read.	1
